# Supplementary material for: Spatial and Quantitative Analysis of Tumor-Associated Macrophages: Intratumoral CD163-/PD-L1+ TAMs as a Marker of Favorable Clinical Outcomes in Triple-Negative Breast Cancer
Source: Int J Mol Sci. 2022 Oct 31;23(21):13235. doi: 10.3390/ijms232113235 (PMC9656504; doi:10.3390/ijms232113235)

**Supplementary Figure S1. Number of positive cells for single and multiple markers of stromal TAMs (sTAMs), immune modification, and B-cell markers stratified by ER/PR, HER2, and TN subtypes.**

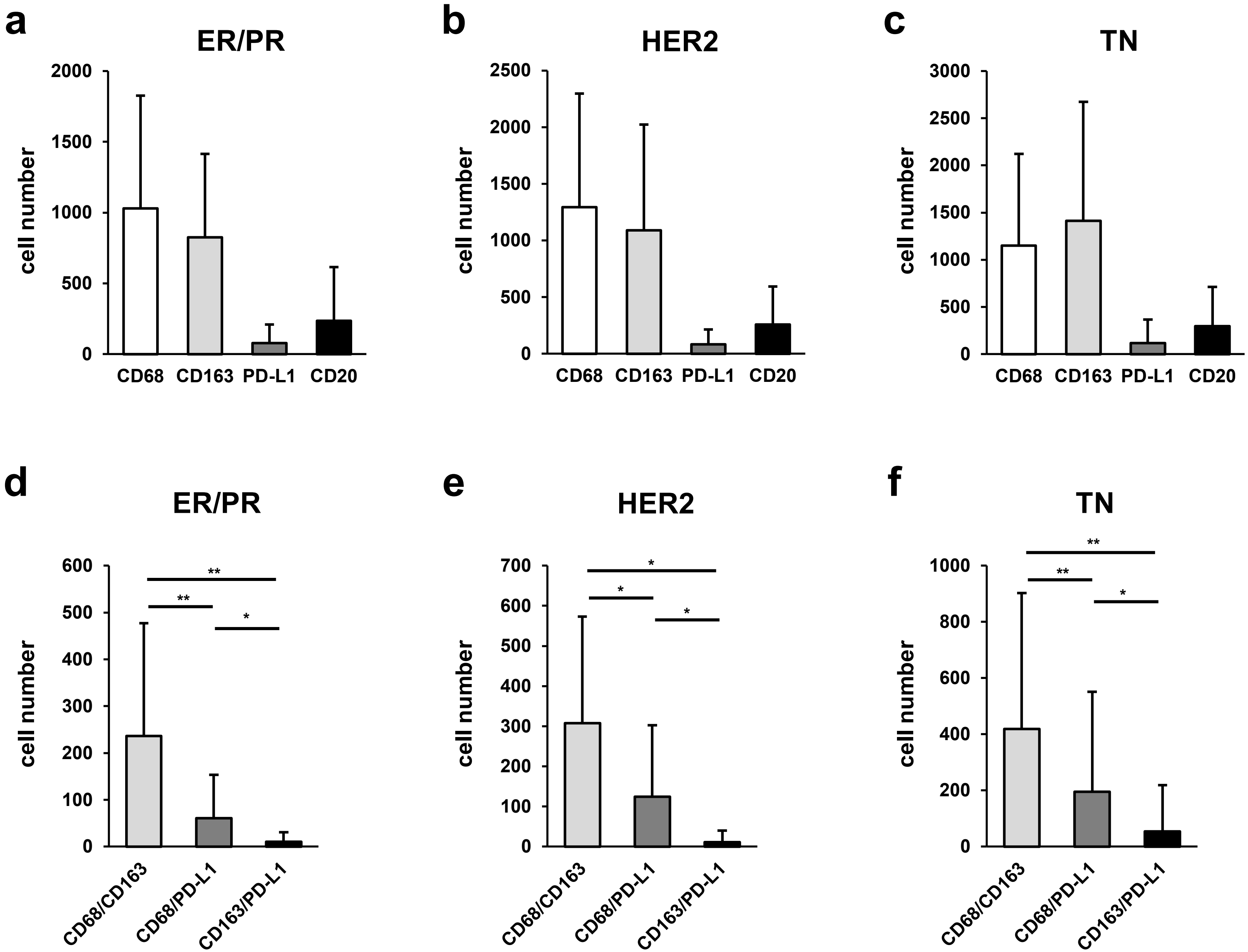

Supplement: Supplementary file 1 [file ijms-23-13235-s001.zip › Supplementary Figure S1.pdf]
