# Supplementary material for: Spatial and Quantitative Analysis of Tumor-Associated Macrophages: Intratumoral CD163-/PD-L1+ TAMs as a Marker of Favorable Clinical Outcomes in Triple-Negative Breast Cancer
Source: Int J Mol Sci. 2022 Oct 31;23(21):13235. doi: 10.3390/ijms232113235 (PMC9656504; doi:10.3390/ijms232113235)

Supplementary Figure S3. Kaplan–Meier analysis for recurrence-free survival categorized by the number of stromal and intratumoral TAMs (sTAMs and iTAMs), immune modification, and B-cell-marker-positive cells in triple-negative cases.

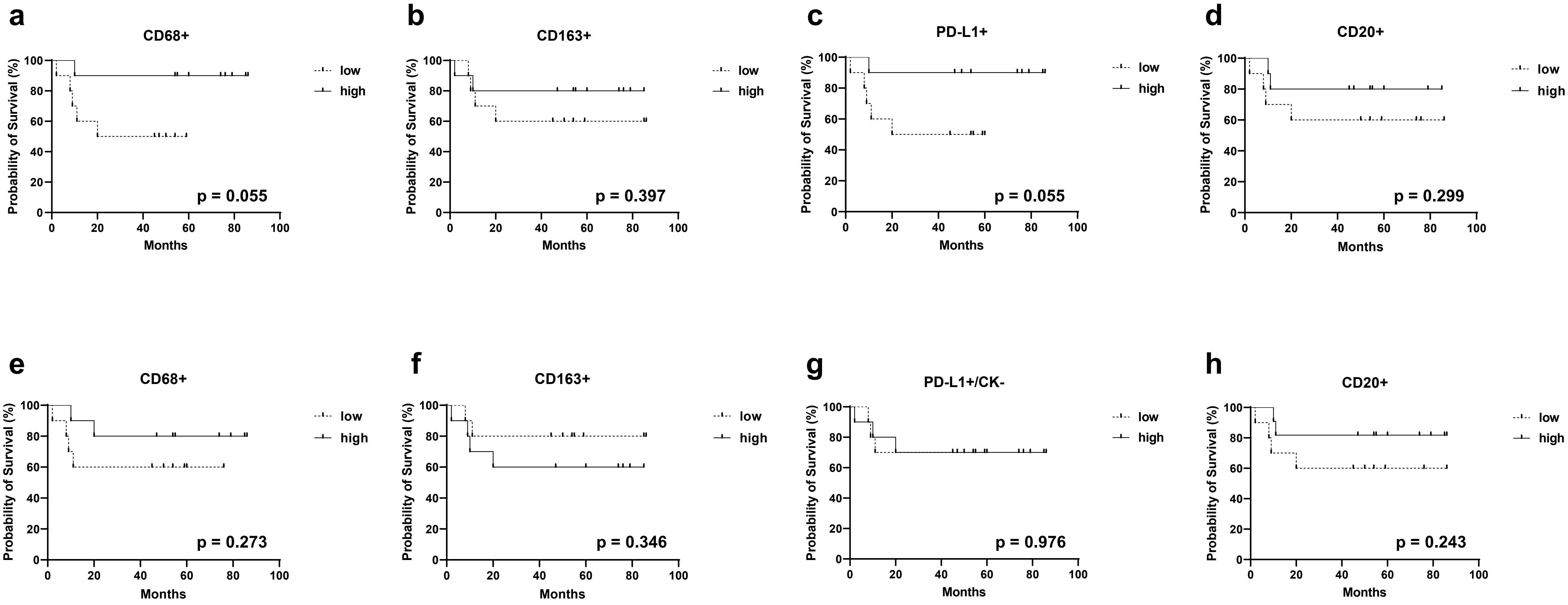

Supplement: Supplementary file 1 [file ijms-23-13235-s001.zip › Supplementary Figure S3.pdf]
