# Supplementary material for: Spatial and Quantitative Analysis of Tumor-Associated Macrophages: Intratumoral CD163-/PD-L1+ TAMs as a Marker of Favorable Clinical Outcomes in Triple-Negative Breast Cancer
Source: Int J Mol Sci. 2022 Oct 31;23(21):13235. doi: 10.3390/ijms232113235 (PMC9656504; doi:10.3390/ijms232113235)
Supplement: Supplementary file 1 [file ijms-23-13235-s001.zip › Supplementary Table S1.pdf]

Supplementary Table S1. Proportions of sTAMs classified by single marker.

| Marker | Subtype | Min(%) | Median(%) | Max(%) | SD |
|---|---|---|---|---|---|
| CD68 | ER/PR | 1 | 17.4 | 98.8 | 17.2 |
| | HER2 | 4.8 | 20.4 | 82.9 | 17.8 |
| | TN | 0.4 | 17 | 63.4 | 14.6 |
| CD163 | ER/PR | 0.3 | 15.5 | 50.9 | 10.5 |
| | HER2 | 1.9 | 15.7 | 63.6 | 12.6 |
| | TN | 2.2 | 20.5 | 72.4 | 14.6 |
| PDL1 | ER/PR | 0 | 0.6 | 22.6 | 3.3 |
| | HER2 | 0 | 0.4 | 14.7 | 2.5 |
| | TN | 0 | 0.5 | 16 | 3.4 |
| CD20 | ER/PR | 0 | 1.3 | 36.4 | 7.3 |
| | HER2 | 0 | 3.9 | 27.5 | 5.5 |
| | TN | 0 | 2.4 | 31.7 | 6.4 |
