# Supplementary material for: Spatial and Quantitative Analysis of Tumor-Associated Macrophages: Intratumoral CD163-/PD-L1+ TAMs as a Marker of Favorable Clinical Outcomes in Triple-Negative Breast Cancer
Source: Int J Mol Sci. 2022 Oct 31;23(21):13235. doi: 10.3390/ijms232113235 (PMC9656504; doi:10.3390/ijms232113235)
Supplement: Supplementary file 1 [file ijms-23-13235-s001.zip › Supplementary Table S2.pdf]

Supplementary Table S2. Proportions of sTAMs classified by multiple markers.

Marker	Subtype	Min(%)	Median(%)	Max(%)	SD
CD68/CD163	ER/PR	0	3.9	38.6	5.1
	HER2	0.4	4.3	16.5	4.2
	TN	0	5.4	33.7	6.3
CD68/PD-L1	ER/PR	0	0.7	13.6	1.9
	HER2	0	1	12.4	2.5
	TN	0	1.4	23	4.2
CD163/PD-L1	ER/PR	0	0.1	2.4	0.4
	HER2	0	0	2.9	0.4
	TN	0	0.1	12.8	2
