# Supplementary material for: Spatial and Quantitative Analysis of Tumor-Associated Macrophages: Intratumoral CD163-/PD-L1+ TAMs as a Marker of Favorable Clinical Outcomes in Triple-Negative Breast Cancer
Source: Int J Mol Sci. 2022 Oct 31;23(21):13235. doi: 10.3390/ijms232113235 (PMC9656504; doi:10.3390/ijms232113235)
Supplement: Supplementary file 1 [file ijms-23-13235-s001.zip › Supplementary Table S3.pdf]

Supplementary Table S3. Proportions of iTAMs classified by single markers.

Marker	Subtype	Min(%)	Median(%)	Max(%)	SD
CD68	ER/PR	0	4	62.1	11.6
	HER2	0	7	88.3	18.6
	TN	0	4.6	65.8	18.2
CD163	ER/PR	0.1	1.3	14	3.3
	HER2	0	1.9	41.7	7.5
	TN	0	2.7	70.3	13.4
PDL1	ER/PR	0	1	57.6	7.4
	HER2	0	4.1	66.8	14.3
	TN	0	4.4	97.8	25.2
CD20	ER/PR	0	0.1	1.9	0.6
	HER2	0	0.3	15.8	2.3
	TN	0	0.1	12.4	2.6
