# Supplementary material for: Spatial and Quantitative Analysis of Tumor-Associated Macrophages: Intratumoral CD163-/PD-L1+ TAMs as a Marker of Favorable Clinical Outcomes in Triple-Negative Breast Cancer
Source: Int J Mol Sci. 2022 Oct 31;23(21):13235. doi: 10.3390/ijms232113235 (PMC9656504; doi:10.3390/ijms232113235)
Supplement: Supplementary file 1 [file ijms-23-13235-s001.zip › Supplementary Table S4.pdf]

Supplementary Table S4. Proportions of iTAMs classified by multiple markers.

Marker	Subtype	Min(%)	Median(%)	Max(%)	SD
CD68/CD163	ER/PR	0	0.1	7.2	1.1
	HER2	0	0.3	8.4	1.7
	TN	0	0.2	11.9	2.3
CD163/PD-L1	ER/PR	0	0.2	17.9	2.5
	HER2	0	0.6	32.8	6.1
	TN	0	1.2	61.7	12
CD68/PD-L1	ER/PR	0	0.2	25.4	3.6
	HER2	0	0.9	51	7.8
	TN	0	0.6	26.6	6.5
