# Supplementary material for: Spatial and Quantitative Analysis of Tumor-Associated Macrophages: Intratumoral CD163-/PD-L1+ TAMs as a Marker of Favorable Clinical Outcomes in Triple-Negative Breast Cancer
Source: Int J Mol Sci. 2022 Oct 31;23(21):13235. doi: 10.3390/ijms232113235 (PMC9656504; doi:10.3390/ijms232113235)
Supplement: Supplementary file 1 [file ijms-23-13235-s001.zip › Supplementary Table S5.pdf]

Supplementary Table S5. Proportions of cancer cells among breast cancer subtypes

Marker	Subtype	Min(%)	Median(%)	Max(%)	SD
CK+PDL1+HLA-	ER/PR	0	0	0	0
	HER2	0	0	0	0
	TN	0	0	0.4	0
CK+PDL1-HLA+	ER/PR	2.8	72	92.7	19.8
	HER2	16.7	69.4	91.1	18.1
	TN	0.2	53.5	85.9	24.2
CK+PDL1+HLA+	ER/PR	0	0	6.2	0.8
	HER2	0	0.1	5.4	0.9
	TN	0	0.1	22.3	4.2
