# Supplementary material for: Spatial and Quantitative Analysis of Tumor-Associated Macrophages: Intratumoral CD163-/PD-L1+ TAMs as a Marker of Favorable Clinical Outcomes in Triple-Negative Breast Cancer
Source: Int J Mol Sci. 2022 Oct 31;23(21):13235. doi: 10.3390/ijms232113235 (PMC9656504; doi:10.3390/ijms232113235)
Supplement: Supplementary file 1 [file ijms-23-13235-s001.zip › Supplementaryü@Figure Legend.pdf]

Supplementary Figure S1.
The average number of positive cells for single sTAM markers in each subtype (a) ER/PR, (b) HER2, and TN and multiple sTAM markers in each subtype (d) ER/PR (* and ** indicate $p = 0.002$ and $p < 0.001$, respectively), (e) HER2 (* indicates $p < 0.001$), and (f) TN (* and ** indicate $p = 0.002$ and $p < 0.001$, respectively) per 1 mm^2 at 5 locations; *p*-values were calculated using one-way ANOVA test and Tukey's multiple comparison test. Error bars represent standard deviation.

Supplementary Figure S2.
The average number of positive cells for single iTAM markers in each subtype (a) ER/PR, (b) HER2, and (c) TN and multiple iTAM markers in each subtype (d) ER/PR+ (* and ** indicate $p = 0.010$ and $p < 0.001$, respectively) , (e) HER2 (* and ** indicate $p = 0.029$ and $p = 0.002$, respectively), and (f) TN (* indicates $p = 0.001$) per 1 mm^2 at 5 locations; *p*-values were calculated using one-way ANOVA test and Tukey's multiple comparison test. Error bars represent standard deviation.

Supplementary Figure S3.
Each TAM- and B-cell-marker-positive cell subpopulation was classified into "low" or "high": (a) CD68+ sTAM low, <950 cells; high, ≥950 cells; (b) CD163+ sTAM low, <1000 cells; high, ≥1000 cells; (c) PD-L1+ sTAM low, <50 cells; high, ≥50 cells; (d) CD20+ stromal B-cell low, <200 cells; high, ≥200 cells; (e) CD68+ iTAM low, <300 cells; high, ≥300 cells; (f) CD163+ iTAM low, <200 cells; high, ≥200 cells; (g) PD-L1+ iTAM low, <14 cells; high, ≥14 cells; (h) CD20+ intratumoral B-cell low, <25 cells; high, ≥25 cells.
